# Pescoids and Chimeras to Probe Early Evo-Devo in the Fish *Astyanax mexicanus*

**DOI:** 10.3389/fcell.2021.667296

**Published:** 2021-04-13

**Authors:** Jorge Torres-Paz, Sylvie Rétaux

**Affiliations:** Université Paris-Saclay, CNRS, Institut des Neurosciences Paris-Saclay, Gif-sur-Yvette, France

**Keywords:** *Zic1:GFP* lines, evo-devo, eye, gastruloids, cavefish, *Astyanax mexicanus*, ntl, chimeric embryos

## Abstract

The fish species *Astyanax mexicanus* with its sighted and blind eco-morphotypes has become an original model to challenge vertebrate developmental evolution. Recently, we demonstrated that phenotypic evolution can be impacted by early developmental events starting from the production of oocytes in the fish ovaries. *A. mexicanus* offers an amenable model to test the influence of maternal determinants on cell fate decisions during early development, yet the mechanisms by which the information contained in the eggs is translated into specific developmental programs remain obscure due to the lack of specific tools in this emergent model. Here we describe methods for the generation of pescoids from yolkless-blastoderm explants to test the influence of embryonic and extraembryonic tissues on cell fate decisions, as well as the production of chimeric embryos obtained by intermorph cell transplantations to probe cell autonomous or non-autonomous processes. We show that *Astyanax* pescoids have the potential to recapitulate the main ontogenetic events observed in intact embryos, including the internalization of mesodermal progenitors and eye development, as followed with *zic:GFP* reporter lines. In addition, intermorph cell grafts resulted in proper integration of exogenous cells into the embryonic tissues, with lineages becoming more restricted from mid-blastula to gastrula. The implementation of these approaches in *A. mexicanus* will bring new light on the cascades of events, from the maternal pre-patterning of the early embryo to the evolution of brain regionalization.

## Introduction

Emergent model organisms offer novel possibilities to unravel specific questions related to developmental and cell biology. However, some limitations, inherent to each animal system, often render difficult the implementation of novel methodologies.

*Astyanax mexicanus* species has thrived as an emergent model organism for evolutionary developmental biology studies (Torres-Paz et al., 2018; Jeffery, 2020). Its success in this field is due to the existence of two markedly different eco-morphotypes within the same species. *A. mexicanus* comprises river-dwelling fish populations, “surface fish,” and several populations adapted to the life in caves in complete and permanent darkness, “cavefish.” During cave adaptation, striking morpho-functional modifications occurred. Compared to the surface fish, the cave-adapted morphs have completely lost their eyes and pigmentation. In addition, some constructive traits have also emerged such as larger olfactory organs and more numerous facial neuromasts and taste buds, which probably contribute to a sensory compensation for the loss of the visual system (Varatharasan et al., 2009; Yoshizawa et al., 2010; Bibliowicz et al., 2013; Hinaux et al., 2016; Blin et al., 2018). Most of the morphological differences observed in the nervous system of *A. mexicanus* morphotypes have an early embryonic origin (Yamamoto and Jeffery, 2000; Pottin et al., 2011; Hinaux et al., 2016; Rétaux et al., 2016). In fact, recent evidence has shown that maternal determinants, present in the oocyte before fertilization and before zygotic developmental programs are initiated, have an important contribution to later phenotypes (Ma et al., 2018, 2020; Torres-Paz et al., 2019). Indeed, any differential composition of maternal determinants in the eggs is susceptible to lead to changes in early developmental events, such as activation of the zygotic genome, embryonic patterning and establishment of signaling centers, thus affecting later ontogenetic processes.

In fish, the extraembryonic yolk cell (of maternal origin) is an important source of inductive signals that pattern the overlying blastoderm, the embryo proper. Asymmetric segregation of maternal determinants leads to the induction of the embryonic organizer in the prospective dorsal side of the blastoderm. This symmetry breaking event will lead to localized production of different morphogens, creating gradients of signaling activity within the developing embryo. The integration of these signals by embryonic cells provides them with positional information and instructs them to follow a particular developmental program (Schier and Talbot, 2005). Thus, changes in the information contained in the oocytes, represented by maternally inherited RNAs and proteins, will affect the subsequent sequence of developmental events. Hence, *A. mexicanus* offers a unique model to test the maternal influence on embryonic development. However, methods to assess the effect of signaling centers (embryonic and extraembryonic) and the potential of cells to respond to these signals have not been developed yet in this model.

Here, we describe the implementation in *A. mexicanus* of methods used in well-established fish models to probe mechanisms of cell/tissue specification during early embryogenesis. First, we have adapted a recent method of embryonic explant culture developed in zebrafish and known as “pescoids” (i.e., gastruloids derived from fish embryonic cells) to grow the blastoderm after removal of the extraembryonic yolk cell (Fulton et al., 2020; Schauer et al., 2020). Under these conditions of altered embryonic geometry and physical constraints, the pescoids are able to recapitulate the main processes observed in intact embryos such as symmetry breaking, germ layer specification and elongation. In addition to previous reports on pescoids, here we found clear indications of mesoderm internalization and eye development. These pescoids will allow comparative analyses of gene expression in *Astyanax* morphs in the absence of yolk-derived signaling. Second, we have set up the conditions to efficiently achieve inter-morph cell transplantations at matching stages during early embryogenesis. Cell grafting have been widely performed in zebrafish embryos to test cell autonomy and potential during development, as well as to dissect lineage and timing aspects during cell specification. Grafts have also been performed between distinct species such as zebrafish and medaka to study developmental heterochronies (Fuhrmann et al., 2020). In *A. mexicanus*, inter-morphs cell transplantation will allow asking similar questions in a micro-evolutionary context. Further, the implementation of these methodologies to generate pescoids and chimeric embryos in *A. mexicanus* will help to explore the effect of embryonic and extraembryonic signals in cell decisions during early development.

## Materials and Methods

### Fish and Embryo Collection

Our *A. mexicanus* colonies were obtained in 2004 from the Jeffery laboratory at the University of Maryland, College Park, United States. The surface fish stock derives from rivers in Texas, United States and the cavefish from the Pachón cave in San Luis Potosi, Mexico. Fish were since then maintained on a 12:12 hr light:dark cycle at a temperature of 22°C for cavefish and 26°C for surface fish. Reproductions were induced every other week by changes in water temperature: for cavefish temperature was increased to 26°C, and for surface fish temperature was decreased to 22°C during 3 days followed by an increase to 26°C (Elipot et al., 2014). Fish from both morphotypes spawn regularly the first and second days following the increase in temperature. Here, embryos were obtained exclusively by *in vitro* fertilization in order to ascertain synchronous early development. Embryo dechorionation was performed by enzymatic treatment with Pronase 1 mg/mL (Sigma) and embryos were maintained in Embryo Medium (EM) at 24°C. Surface and Pachón cavefish *zic1:GFP* transgenic lines used here were generated previously in the lab (Devos et al., 2019). Animals were treated according to the French and European regulations for handling of animals in research. SR’s authorization for use of *Astyanax mexicanus* in research is 91–116. This work did not necessitate a protocol authorization number from the Paris Centre-Sud Ethic Committee. The animal facility of the Institute received authorization 91272105 from the Veterinary Services of Essonne, France, in 2015.

### Generation of Chimeric Embryos by Cell Transplantations

Donor embryos were injected at the 1-cell stage with 3–5 nL of 1% Dextran-FITC 10,000 MW (Molecular Probes) and 0.05% Phenol Red (to see the solution) using a FemtoJet (Eppendorf). Glass pipettes for microinjection and cell transplantation were prepared on a Narishige PN-30 puller using borosilicate glass capillary (GC100F15 Harvard Apparatus LTD and B120-69-10 WPI, respectively). Microinjection pipettes were sealed at the tip and broken for opening at the moment of the injection using forceps. Cell transplantations pipettes were prepared in advance, the tip was broken at the desired internal diameter (15–30 μm) and polished using a Micropipette grinder (Narishige EG-44) at an angle of 35° in order to create a smooth needle-shaped tip. Our cell transplantation system consisted of a holder for the glass pipette (WPI) connected to a 1 mL syringe by a Teflon tubing (Narishige). Under a fluorescent dissecting microscope, labeled donor cells were aspirated into the tip of the glass pipette filled with EM, and 3–12 cells expelled into the host embryo with gentle pressure. Host embryos were let to develop in EM until fixation. In this study, isotopic and isochronic intermorphs grafts were performed (Surface animal pole cells into Cave animal pole, either at blastula or gastrula stages). Of note, targeting of cells to specific regions should be significantly more precise at gastrula stages, with the appearance of the organizer as a reference and morphological landmark for the dorsal side. *In vivo*, dextran-FITC labeled cells in grafts could be visualized at least up to 1 dpf (not tested later in this study).

### Generation of Pescoids

**FIGURE 1:**
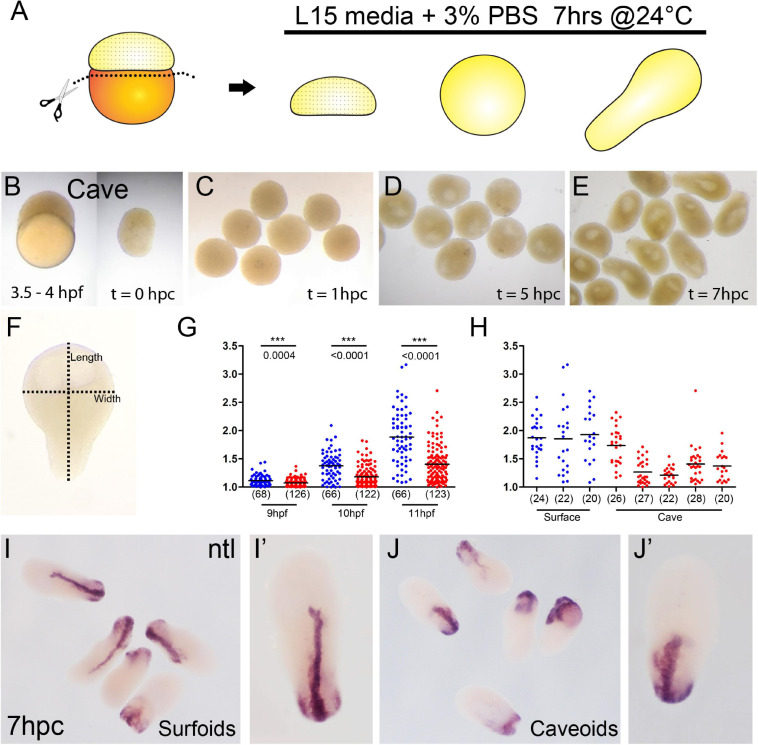
Generation of pescoids in *A. mexicanus*. Procedure for the generation of *Astyanax* pescoids **(A)**. Intact cavefish embryo at 1K-cell stage [**(B), left**] and after yolk removal [**(B), right**]. Development of caveoids after 1, 5, and 7 hpc [**(C–E)**, respectively]. Measurement of the length and width on a pescoid to calculate the aspect ratio value as length/width **(F)**. Measurement of aspect ratio of surface (blue) and cave (red) pescoids at 9, 10, and 11 hpf **(G)**. Statistical differences (***) between the two morphs are indicated with the corresponding p values obtained by Mann-Whitney tests at every time point and the numbers in parentheses indicate the number of pescoids analyzed **(G)**. Aspect ratio measurement at 11 hpf in pescoids derived from individual females shows variability in cavefish (3 different females for surfoids, 5 different females for caveoids) **(H)**. Black lines indicate the mean value **(G,H)**. Expression of *ntl* in surfoids **(I,I′)** and caveoids **(J,J′)** after 7 hpc.

*Astyanax mexicanus* pescoids were produced following a recent description in zebrafish (Fulton et al., 2020; Figure 1A). Briefly, at the 512-1K cell stage the yolk was carefully removed from embryos using eyebrows knives. Blastoderm explants were cultured until the corresponding 11 h post-fertilization (hpf) at 24°C in L15 medium (Gibco) supplemented with 3% Fetal Bovine Serum (FBS, Biosera). For cultures maintained for longer than 7 h Penicillin-Streptomycin were added to the culture media (1x dilution, P4333, Sigma).

### Histology and Imaging

Colorimetric ISH was performed as previously described (Pottin et al., 2011). Digoxigenin-labeled riboprobe was prepared using PCR products as templates. *no-tail* (*ntl*) cDNA was obtained from our ESTs library (accession number ARA0ABA99YL22). Procedure for revelation of FITC-labeled donor cells combined with fluorescent ISH was adapted from our ISH protocol (Alié et al., 2018). Following the hybridization with the Digoxigenin-labeled ISH probe, embryos were first processed for the revelation of the Dextran-FITC grafted cells: they were incubated for 1 h in blocking solution (Tris 0.1M pH 7.5, NaCl 150 mM, Tween-20 0.1 and 5% blocking reagent Roche) and then with POD-conjugated anti-FITC antibody (11426346910; Roche, 1/400) diluted in blocking solution overnight. Embryos were washed in PBS/Tween 0.1% (PBST) 10 times for 10 min each time and incubated for 30 min at room temperature with TAMRA-tyramide 1/1000. Peroxidase activity was activated by H2O2 (0.003%, Sigma) for 1 h and samples were washed again 10 times for 10 min in PBST. Revelation of the dig-labeled ISH probe was then performed using an anti-Digoxigenin antibody coupled to POD (11207733910; Roche, 1/400) and revealed using a FITC-tyramide (1/400). After several washes in PBST embryos were stained with DAPI (10236276001, Sigma) at a final concentration of 1 mg/ml, overnight at 4°C, and washed in PBS before dissection and mounting (Vectashield, Vector Laboratories).

Immunohistochemistry was performed as previously described (Blin et al., 2018) using a primary anti-GFP antibody at a dilution 1/500 (GFP-1020, Aves Labs) and a secondary Alexa Fluor 488-coupled antibody at a dilution 1/500 (Anti-chicken, A-11039, Invitrogen). Fluorescently labeled embryos were counterstained with DAPI. Embryos stained by colorimetric ISH were imaged on a Nikon AZ100 multizoom macroscope. Confocal acquisitions were done on a Leica-SP8 confocal microscope using the Leica Application Suite software. Images processing and quantifications were done on Fiji and statistical tests (Mann-Whitney non-parametric tests) were performed on GraphPad Prism.

## Results and Discussion

### Generation of Astyanax Pescoids

Recent advances in the field of gastruloids have highlighted the robustness of animal development and the key steps taking place during this process. In zebrafish pescoids (yolk-less blastoderm explants) the main aspects of early development observed in intact embryos are recapitulated. Symmetry breaking, axis elongation and neural specification occur despite the absence of extraembryonic signaling (Fulton et al., 2020; Schauer et al., 2020). In fish, these events are controlled maternally (Marlow, 2020; Solnica-Krezel, 2020) and *A. mexicanus* with its two morphotypes has become an excellent model to challenge the role of maternal determinants in embryogenesis (Ma et al., 2018, 2020; Torres-Paz et al., 2019).

Pescoids derived from embryos of both *Astyanax* surface and cavefish embryos (surfoids and caveoids, respectively) developed similarly to those described in zebrafish. After removal of the vitellus at the 256-1K cell stage (Figure 1B), blastoderm explants sealed the wound and became rounded during the first hour post culture (hpc, equivalent to 5–6 hpf, Figure 1C). Then, during the next 3–4 h in culture a cavity was formed (8–9 hpf, Figure 1D), which may correspond to a “blastocoel” (Schauer et al., 2020). After 5 hpc (9 hpf), the first signs of axis elongation were observed in surfoids and caveoids (Figures 1E–G). The shape of the elongated pescoids was asymmetrical and pear-shaped, with a narrow tip at one end and a larger rounded form at the opposite extremity. The extent of elongation was quantified by calculating the aspect ratio (length/width, Figure 1F) at three timepoints up to the end of the culture at stages corresponding to tailbud/early somitogenesis (9, 10, and 11 hpf, Figures 1F–H). Both surfoids and caveoids underwent significant elongation during this time window. At the three stages analyzed, however, we found reduced elongation of caveoids compared to surfoids (Figure 1G). Moreover, we observed inter clutch variability in the extent of elongation in pescoids derived from different cavefish females, but much less so between different surface fish females, and always with higher mean length values in surface-derived pescoids as shown at 11 hpf (Figure 1H). These results are counterintuitive considering our previous observations of more precocious axial mesoderm extension in cavefish compared to surface embryos during gastrulation (Torres-Paz et al., 2019). Reduced elongation in cavefish pescoids may originate from deficiencies in the regulative properties of embryonic cells and/or the maternal pre-patterning at the blastula stages (Fulton et al., 2020). Insights on maternal control of early patterning and morphogenesis will come from pescoids obtained from F1 reciprocal hybrid embryos (Ma et al., 2018; Torres-Paz et al., 2019).

**FIGURE 2:**
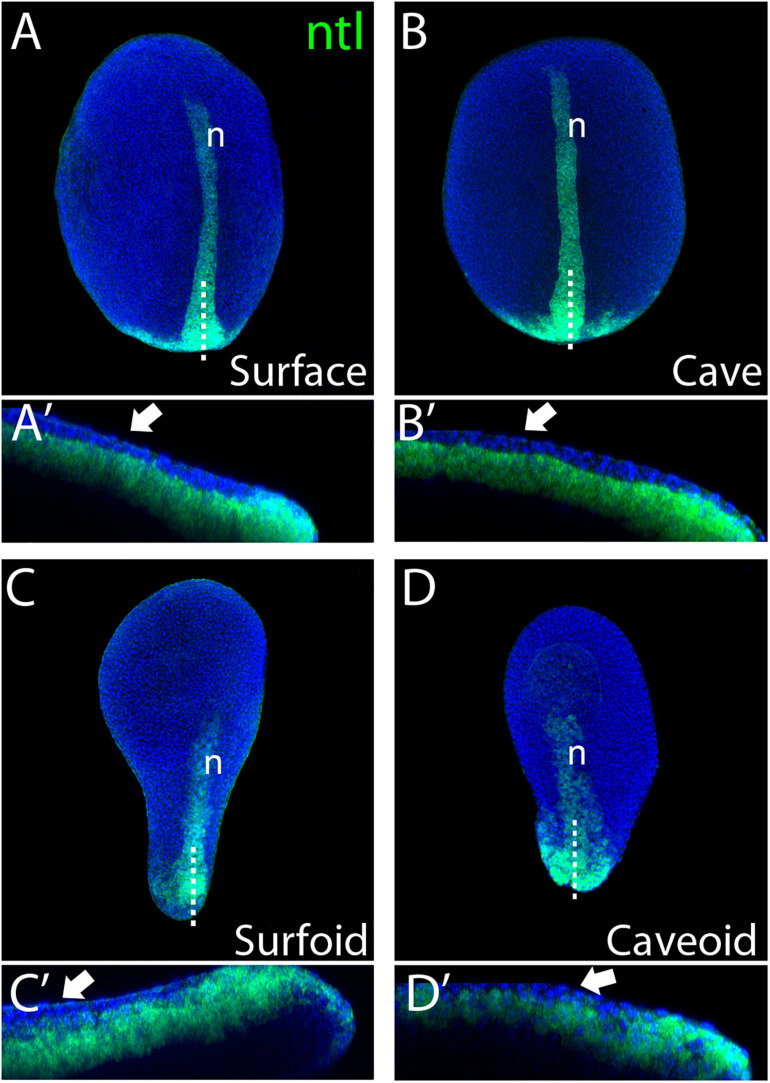
Internalization of mesoderm in *Astyanax* pescoids. Expression of *ntl* in intact surface and cavefish embryos at 10 hpf [**(A,B)**, respectively], and in surfoid and caveoid after 7 hpc [**(C,D)**, respectively]. Embryos are oriented in dorsal views, anterior to the top. For each panel, bottom images **(A’–D’)** are confocal reconstructions at the indicated level (dotted lines), anterior to the left and dorsal to the top. The white arrows indicate ectodermal cells overlying *ntl*-expressing mesodermal cells and n indicates the notochord.

To get further insights into the elongation process, we studied the expression of the mesodermal marker *ntl* in *Astyanax* pescoids and found a pattern strikingly reminiscent of the developing notochord observed in intact fish embryos (Figures 1I,J). Microscopic observations suggested that cells expressing *ntl* had been internalized, an aspect of fish gastruloids that may have been overlooked. We thus compared the expression of *ntl* in confocal acquisitions after fluorescent ISH in intact embryos at tailbud stage and in pescoids at equivalent stages (Figure 2). In confocal reconstructed sections of pescoids and control embryos, we observed similar organization of *ntl* expressing cells, always underneath an overlying layer of superficial cells, that we interpreted as ectoderm cells (Figure 2, arrows). Thus, these data confirm a conserved internalization process of axial mesoderm in explants despite the absence of vitellus. These observations highlight the robustness of cellular processes during vertebrate gastrulation.

Studies in zebrafish have shown that asymmetric translocation of maternal determinants in the yolk leading to the dorsal determination occur as early as the 16–32 cell stage (Jesuthasan and Strähle, 1997). The activation of the zygotic genome, a process dependent on the maternal transcriptomic machinery, starts around the 64 cell stage (Chan et al., 2019), whereas clear zygotic transcription is observed from the 512 cell stage. Concurrently, at this stage, called the mid-blastula transition, embryonic cell cycles become asynchronous and the extra-embryonic yolk syncytial layer (YSL) is formed at the interphase between the yolk and the blastoderm (Kimmel et al., 1995). Similarly to zebrafish, in *Astyanax* the mid-blastula transition takes place around the 512-1K cell stage (Hinaux et al., 2011). Here, the explant experiments were performed between the 128-1K cell stage, i.e., the end of the maternal-to-zygotic transition, thus it must be taken into account that maternally-derived pre-patterning already exists in the cultured blastoderms. Consequently, in order to gain insights into the temporal sequence of maternal patterning events, the generation of even earlier explants must be considered. The comparative analysis of earlier explants in the two *Astyanax* morphs will allow dissecting precisely the timing and the impact of the maternal contributions to developmental evolution. Recently, it was shown in zebrafish that the extra-embryonic YSL layer does not form properly in yolkless-explants, yet correctly developing pescoids can be obtained even from very precocious 64 cell stage embryos (Schauer et al., 2020). This indicates that pescoids can develop into embryo-like structures in the absence YSL-derived signals (Rodaway et al., 1999; Chen and Kimelman, 2000). Conversely, animal caps explants are able to develop into structures similar to pescoids only if Nodal and downstream planar cell polarity signaling pathways are active (Williams and Solnica-Krezel, 2020), indicating that Nodal activity in pescoids must come from marginal cells. Nodal signaling is necessary for the induction of endomesodermal fates at the blastoderm margin (Schier et al., 1997; Vopalensky et al., 2018). Visual inspection of the mesodermal marker *ntl* expression in our pescoids at the different states of elongation clearly showed that the point where *ntl*-expressing cells are internalized corresponded to the marginal zone, where the wound closed (not shown). Thus, the wounded margin in the blastoderm explants would be topologically and functionally equivalent to the blastopore in intact embryos, being both the source of Nodal signaling and the point where endomesoderm is internalized. Given the previously described differences in organizer formation and mesoderm internalization between the two *Astyanax* morphs embryos (Torres-Paz et al., 2019), pescoids will prove powerful tools to study the origin and outcomes of these processes.

### Eye Development in *Astyanax* Pescoids

**FIGURE 3:**
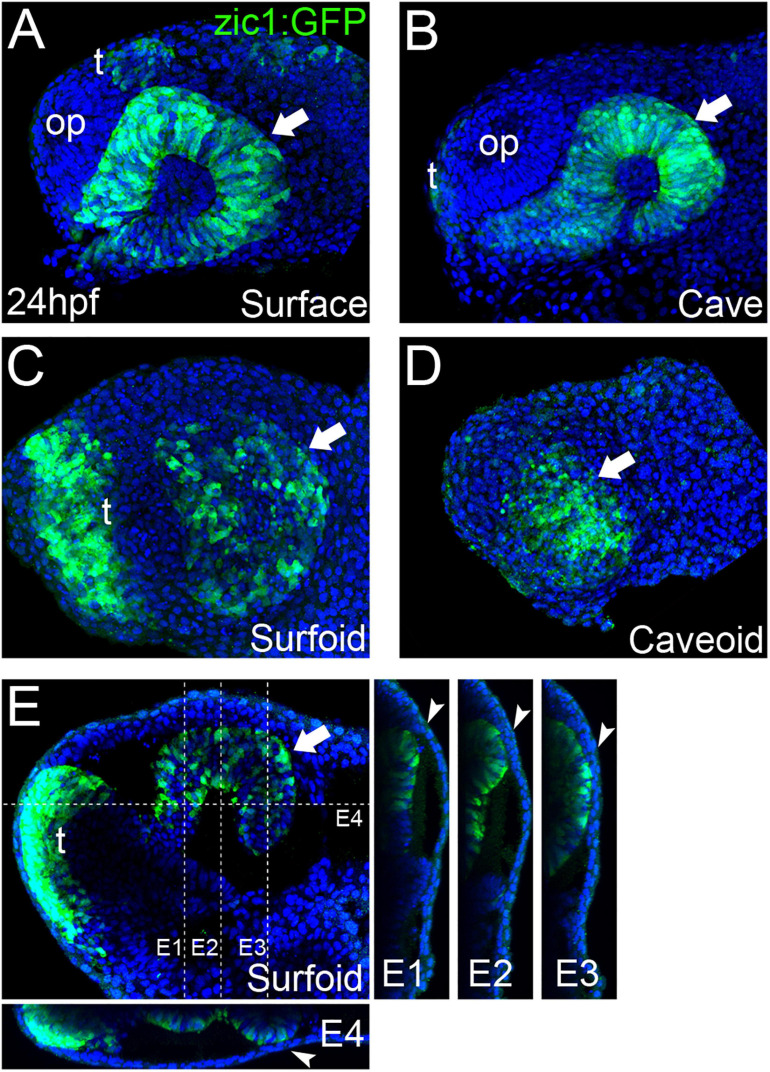
Eye development in *Astyanax* pescoids. GFP-reporter expression in *zic1:GFP* transgenic surface and cavefish intact embryos [**(A,B)**, respectively], and surfoid **(C,E)** and caveoid **(D)** at 24 hpf. Embryos and pescoids are oriented in lateral views, anterior to the left. Arrows indicate the GFP-expressing optic tissue; op, olfactory placode; t, telencephalon. Confocal reconstructions at the indicated levels in **(E)** are shown **(E1–E4)**. Arrowheads **(E1–E4)** indicate the contact zone between neural and non-neural ectoderm.

Our observations of axial elongation and the overtly normal formation of the notochord in pescoids (Figure 2) made us wonder if eye development could also occur in these yolkless explants. We recently generated *zic1:GFP* transgenic knock-in reporter lines in both *Astyanax* morphotypes in order to visualize eye morphogenesis (Devos et al., 2019). We took advantage of these transgenic fish to evaluate directly eye development in pescoids. Blastoderm explants from *zic1:GFP* embryos were let to develop *in vitro* until the equivalent of 24 hpf, i.e., when the optic cups are formed in surface and cave embryos (Figures 3A,B). Despite some “body axes” malformations in these explants (not shown), we observed GFP reporter expression in discrete regions within the prospective pescoid head (Figures 3C–E). Strikingly, we were able to identify distinct GFP expression domains that we interpreted as corresponding to the optic (arrows, Figure 3) and anterior telencephalic (“t,” Figure 3) *zic1-*expressing tissues, respectively, like in intact embryos (Supplementary Figure 1). We cannot rule out that, in some pescoids, the separation of the telencephalic and optic GFP expressing cells could be perturbed and result in a single GFP positive domain containing intermingled cells with mixed identities (Figure 3D). Further studies analyzing cell movements during pescoids “neurulation” will be needed to address this question.

In embryos, the size of the optic cup is known to be smaller in cavefish (Yamamoto and Jeffery, 2000; Alunni et al., 2007). We observed the same tendency in the *zic1:GFP* transgenics (Figures 3A,B, Surface = 23,557 ± 1,336 μm^2^, *n* = 4, mean ± standard deviation, n; Cave = 15,169 ± 2,395 μm^2^, *n* = 3; −35% in cavefish embryos; see also Devos et al., 2019). In pescoids, the extension of the optic domain delimited by reporter expression varied importantly, when present and identifiable, in the two morphotypes, but it was smaller in caveoids (Figures 3C–E, Surfoids = 15,998 ± 6,329 μm^2^, *n* = 3; Caveoids = 11,355 ± 4,460 μm^2^, *n* = 4; −30% in caveoids). Thus, on the small sample analyzed the difference in size of the optic tissue mirrors the situation in whole embryos, suggesting a conservation of the control of organ size in pescoids. Surprisingly, in two surfoid specimens we even found a high degree of retinal morphogenesis (Figures 3E,E1–E4), with clear indications of epithelial folding (arrow, Figure 3E) and neural tissue contacting adjacent non-neural ectoderm (arrowheads, Figures 3E1–E4). These observations further highlight the robustness of developmental processes occurring in gastruloids.

Eye development starts with the specification of eye precursor cells in the anterior neural plate (Zuber et al., 2003; Cavodeassi et al., 2005), followed by complex morphogenesis processes that involve coordinated movements within the neural plate (Cavodeassi et al., 2005; Rembold et al., 2006; Picker et al., 2009; Kwan et al., 2012; Ivanovitch et al., 2013) that are instructed by midline signaling (Macdonald et al., 1995; García-Calero et al., 2008; Gordon et al., 2018). Our observations of eye-like embryonic tissue in *Astyanax* pescoids demonstrate the potential of gastruloid systems to engage into complex morphogenesis. The wide spectrum of optic phenotypes observed in our pescoids will allow a better understanding of minimal requirements for particular developmental processes and the interdependency of different embryonic tissues during morphogenesis.

### Generation of Chimeric Embryos by Cell Grafting

Cell transplantation methodologies have been widely used in zebrafish to test cell-autonomy during embryogenesis in different experimental contexts (Fauny et al., 2009; Cavodeassi et al., 2013; Giger et al., 2016). *A. mexicanus* with its two eco-morphotypes offers a great opportunity to test autonomy during cell fate specification through intermorph transplantations. Hence, elegant transplantation experiments of the lens from one morph into the optic cup of the other morph at larval stages have revealed the autonomy of the cavefish lens apoptotic process and its triggering role in cavefish eye degeneration (Yamamoto and Jeffery, 2000). Neural crest cell transplantations have been performed as well at 2 dpf to address their role in cavefish pigmentation defect (Yoshizawa et al., 2018). However, the *Astyanax* model has not succeeded yet for early cell transplantations aimed at studying precocious embryogenesis, mainly due to technical challenges that must be circumvented. A major challenge is the simultaneous collection of embryos of both morphotypes at equivalent developmental stage. *A. mexicanus* reproduce in the dark (Simon et al., 2019), thus in order to obtain early developing embryos to work with during the day, we inverted the circadian cycle of fish in a special fish room dedicated to reproduction in our facility. In addition, to obtain early embryos developing synchronously, *in vitro* fertilizations must be performed using ready-to-spawn females. Hence, mating behavior was monitored during the days following the induction of reproductions with a camera in the circadian-inverted fish room illuminated with a dim red-light. Inductions of surface and cavefish were then coordinated in order to find spawning females of the two morphs at the same time.

**FIGURE 4:**
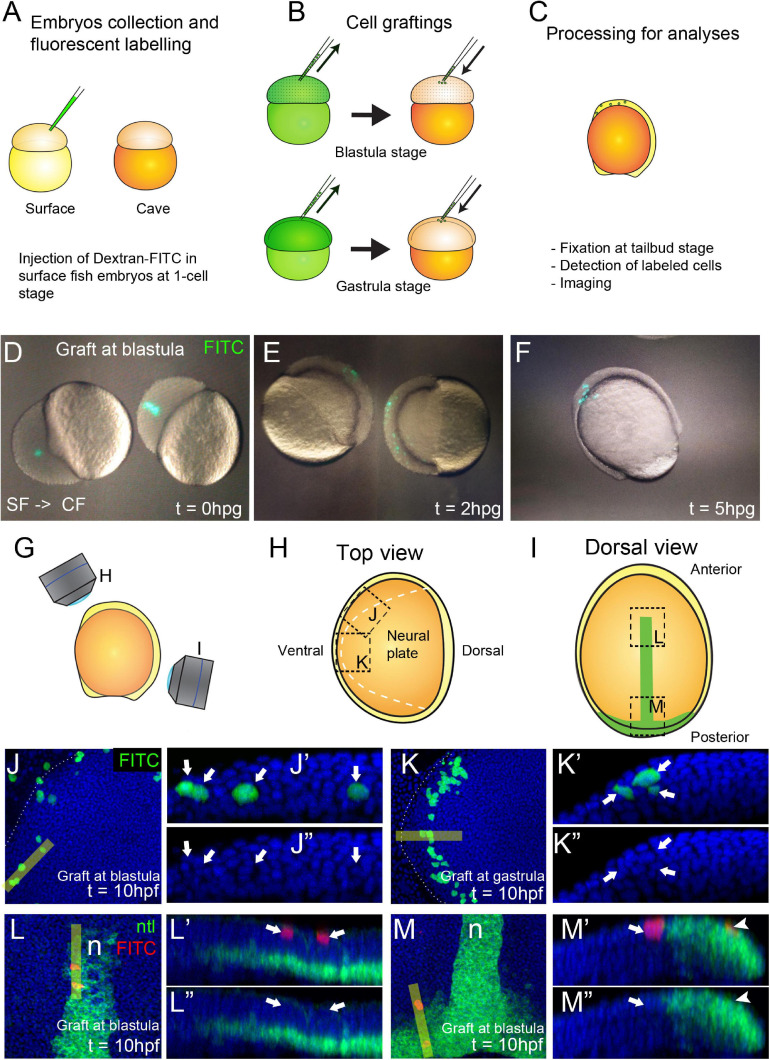
Generation of chimeric embryos in *A. mexicanus* by inter-morph cell transplantation. Procedure for cell grafting between surface and cavefish embryos **(A–C)**. Live cavefish (host) embryos that have been transplanted with FITC-labeled surface cells (green) at the blastula stage, photographed immediately after the grafting [0 hpg, **(D)**], or after 2 hpg **(E)** and 5 hpg **(F)**. Fluorescent labeled embryos at tailbud stage were imaged in a confocal microscope in a view from the top **(G,H)** and from the dorsal side **(G,I)**. Black dashed lines indicate the orientation of the images shown in panels **(J–M)**. Dashed white line indicate the neural plate border **(H,J,K)**. Confocal images of dissected cavefish embryos at 10 hpf at the level of the anterior neural plate **(J, K)**. Labeling in green corresponds to surface fish cells grafted at blastula **(J)** and gastrula **(K)** stages. Respective reconstructed sections were obtained at the levels indicated in yellow bars and grafted cells are indicated with arrows **(J′,J″,K′,K″)**. Confocal images of dissected cavefish embryos at 10 hpf stained for *ntl* [green, **(I,L,M)**], at the level of the anterior notochord **(L)** and the tail bud **(M)**. Labeling in red corresponds to surface fish cells grafted at blastula stage and labeling in greencorresponds to *ntl* expression in the notochord. Respective reconstructed sections were obtained at the levels indicated in yellow bars **(L′,L″,M′,M″)**. Arrows **(L′,L″,M′,M″)** indicate superficial ectodermal grafted cells. Arrowheads **(M′,M″)** indicate a cell in the mesodermal domain expressing *ntl*. Images in panels **(J,K)** are top views, anterior to the left. Panels **(L,M)** are dorsal views, anterior to the top. Reconstructions are oriented with anterior to the left and dorsal on top.

In this work surface fish embryos were labeled with dextran-FITC at the one-cell stage (donors), and cells were grafted isochronically into unlabeled cavefish embryos (hosts) at two developmental time points, the mid-blastula transition (512-2K cell stage) and the onset of gastrulation (30–50% epiboly) (Figures 4A,B). As a source of donor cells we choose the embryonic animal pole, in order to compare to fate maps studies performed in zebrafish and showing that ectodermal precursors (including neurectoderm) arise from this field, whereas endomesodermal precursors derive from more marginal cells (Woo and Fraser, 1997; Schier and Talbot, 2005). After transplantation of fluorescently labeled cells (Figure 4D), embryonic development was observed to occur normally during the following hours post grafting (hpg), with labeled cells integrated in the embryo (Figures 4E,F). After fixation and methanol storage of chimeric embryos, FITC fluorescence in labeled grafted cells was completely lost (not shown), rendering necessary a revelation through immunohistochemistry (Figure 4C). Fluorescent revelation of labeled cells with FITC-coupled tyramide was avoided because an extensive bleed through of fluorescence to channels at lower wavelengths was observed (not shown). Instead, revelation with TAMRA-coupled tyramide, whose fluorescent excitation/emission occurs at higher wavelengths (557 and 583 nm, respectively) than FITC (495 and 521 nm, respectively), did not produce bleed through to lower wavelengths channels (Figures 4G′,G″,H′,H″).

We compared the organization and repartition of grafted cells in host embryos at the two transplantation stages, blastula versus gastrula, and observed clear differences between the two conditions. Clones transplanted at blastula stage were distributed extensively throughout the embryos and in a disorganized and scattered manner. On the other hand, cells transplanted at gastrula stages were restricted in space and in some cases showed clear signs of symmetry (compare Figures 4E,F). Using the positional information and the expression of *ntl* in the notochord as reference (**Figure 4I**), we observed that cells grafted at blastula stages were able to produce both ectodermal (*n* = 15/15 embryos, superficial cells in Figures 4L,L′,L″) and endomesodermal (*n* = 6/15 embryos, *ntl* positive cell in Figures 4M,M′,M″) derivatives in chimeric embryos. On the other hand, grafts performed at gastrula stages gave rise to only ectodermal cells (*n* = 24/24 embryos, Figures 4J,J′,J″,4K,K′,K″). These data were consistent with an expected progressive lineage restriction from mid-blastula to early gastrulation stages. Confocal reconstructions suggested a correct integration of transferred cells in the developing host tissues. Similar results were also observed in reciprocal experiments, i.e., transplants of cavefish donor cells into a surface fish host (not shown). We also found that surface fish embryonic cells were able to differentiate into pigmented cells in a cavefish host (Supplementary Figure 2) confirming the correct integration and differentiation of donor cells in chimeric embryos and illustrating a typical cell-autonomous process.

Intermorph grafting will shed light on the cell autonomy and the effect of the embryonic signaling environment on previously described heterochronies, heterotopies and differences of gene expression levels during development of *Astyanax* morphs (Yamamoto et al., 2004; Pottin et al., 2011; Hinaux et al., 2016; Torres-Paz et al., 2019). The combination of these grafting methods with the use of transgenic reporter lines such as the cavefish and surface fish *zic1:GFP* lines (Devos et al., 2019), will allow the detailed investigation of intrinsic and extrinsic factors implicated in eye specification and degeneration.

## Conclusion

Implementation and optimization of new methods in emergent model systems is fundamental for tackling novel scientific questions. Here we describe the methodology and potential applications of cellular techniques to generate yolk-free pescoids and chimeric embryos in *Astyanax mexicanus*. These methods will allow the characterization of developmental states during cell lineages differentiation in embryogenesis. In addition, these techniques will push forward genomic and cellular approaches to understand the key steps during eye development and degeneration in cavefish.

## Data Availability Statement

The original contributions presented in the study are included in the article/Supplementary Material, further inquiries can be directed to the corresponding author/s.

## Ethics Statement

The animal study was reviewed by the French and European regulations for handling of animals in research. SR’s authorization for use of Astyanax mexicanus in research is 91-116. This work did not necessitate a protocol authorization number from the Paris Centre-Sud Ethic Committee. The animal facility of the Institute received authorization 91272105 from the Veterinary Services of Essonne, France, in 2015.

## Author Contributions

JT-P performedexperiments. JT-P and SR conceived experiments, analyzed data, and wrote the manuscript. Both authors contributed to the article and approved the submitted version.

## Conflict of Interest

The authors declare that the research was conducted in the absence of any commercial or financial relationships that could be construed as a potential conflict of interest.
