# Supplementary material for: Pescoids and Chimeras to Probe Early Evo-Devo in the Fish Astyanax mexicanus
Source: Front Cell Dev Biol. 2021 Apr 13;9:667296. doi: 10.3389/fcell.2021.667296 (PMC8078105; doi:10.3389/fcell.2021.667296)
Supplement: Supplementary file 1 [file Data_Sheet_1.PDF]

Supplementary Material

Supplementary Figures

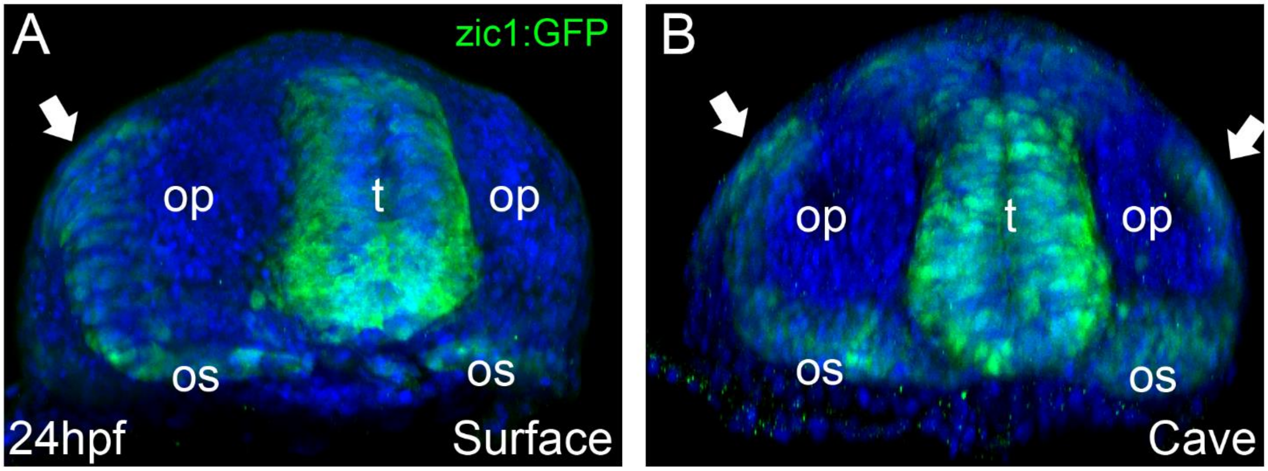

Supplementary Figure 1. GFP expression in the forebrain of transgenic embryos *zic1:GFP*. 3D confocal reconstructions from Surface and Cavefish *zic1:GFP* transgenic embryos (**A and B**, respectively) at 24hpf. Images in frontal view. Arrows indicate the embryonic eyes; op, olfactory placodes; os, optic stalks; t, telencephalon.

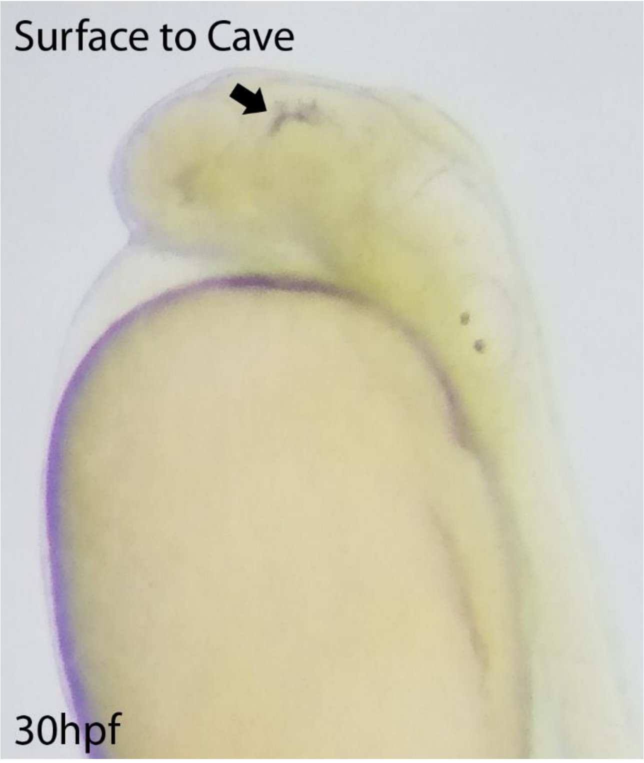

Supplementary Figure 2. Differentiation of pigmented cells in chimeric embryo. Pigmented cell (arrow) derived from surface fish donor cells transplanted into a cavefish embryo at the blastula stage.
